# 97 U Put the Burn in CBRN

**DOI:** 10.1093/jbcr/iraf019.097

**Published:** 2025-04-01

**Authors:** Lauren Caballero, Gustavo Fajardo, Laura Carpenter, Pamela Michelli, Sherrina Richards

**Affiliations:** Warden Burn Center; Warden Burn Center; Warden Burn Center; Orlando Health Regional Medical Center; Warden Burn Center

## Abstract

**Introduction:**

It was not until August of 2020 that the American Nurses Association recognized Burn Nursing as a specialty. That designation sparked the development of the Certified Burn Registered Nurse (CBRN), released in October of 2023.

**Methods:**

A Microsoft Office Forms survey was developed to assess barriers to nurses attempting their CBRN on the Trauma Burn Intensive Care Unit (TBICU) and the Trauma Burn Step-Down Unit (TBSU). The survey had nine questions. The first three questions assessed which demographics. The fourth determined if the nurse had any other certifications. The fifth asked if the nurse participated in review classes. Question six and seven assessed specific barriers. Question eight and nine assessed what the nurse’s opinion was on how barriers could be alleviated.

**Results:**

Of the 82 nurses eligible for the survey, 50 responded. Demographics revealed 48 were a nurse for at least a year and 44 had a Bachelor Science in Nursing or higher. Of all the nurses, 27 already had a different certification.

The top two barriers were the amount of time it took to study and the possibility of failing. Other barriers included the cost, multiple certifications to keep up with, the burn certification being too specific, certifications do not matter professionally, staffing too unstable, not feeling experienced enough, being close to retiring, and lastly someone that had already taken it said it was hard.

Ideas for alleviating these identified barriers were for nurses to be paid more for having the CBRN, to have the exam paid for by our organization, having review classes or study groups at work, improving staffing, getting time off to study, and getting paid to study.

**Conclusions:**

The data showed our leadership team that we have not been clear enough in communicating evidenced by the responses of exam cost and review classes being a barrier.

Since focusing on the CBRN, our organization monetized completing certifications for nursing staff, our burn leadership has emphasized certification by getting certified themselves, and increased communication and dissemination of information improved through the role of the Burn Nurse Mentor. Our future research will focus on the benefits of being CBRN certified and a multi-certified nurse.

**Applicability of Research to Practice:**

According to the National Library of Medicine (2021), Nurse specialty certification serves as a formal acknowledgment of individual nurses’ expertise, documenting their knowledge, skills, and abilities specific to their specialty. This credentialing goes beyond basic education and licensing. Increasing nurse specialty certification is particularly important for hospitals seeking Magnet status, an accreditation that signifies excellence in nursing. By pursuing specialty certification, nurses demonstrate a commitment to professional development and lifelong learning.

**Funding for the Study:**

N/A

